# Design of a stable ethanologenic bacterial strain without heterologous plasmids and antibiotic resistance genes for efficient ethanol production from concentrated dairy waste

**DOI:** 10.1186/s13068-023-02298-z

**Published:** 2023-04-01

**Authors:** Lorenzo Pasotti, Davide De Marchi, Michela Casanova, Angelica Frusteri Chiacchiera, Maria Gabriella Cusella De Angelis, Cinzia Calvio, Paolo Magni

**Affiliations:** 1 Department of Electrical, Computer and Biomedical Engineering, University of Pavia, Via Ferrata 5, 27100 Pavia, Italy; 2 Centre for Health Technologies, University of Pavia, Via Ferrata 5, 27100 Pavia, Italy; 3 Department of Public Health, Experimental and Forensic Medicine, University of Pavia, Via Forlanini 8, 27100 Pavia, Italy; 4 Department of Biology and Biotechnology, University of Pavia, Via Ferrata 9, 27100 Pavia, Italy

**Keywords:** *Escherichia coli*, Bioethanol, Concentrated whey permeate, Fermentation, Metabolic engineering, Chromosomal integration

## Abstract

Engineering sustainable bioprocesses that convert abundant waste into fuels is pivotal for efficient production of renewable energy. We previously engineered an *Escherichia coli* strain for optimized bioethanol production from lactose-rich wastewater like concentrated whey permeate (CWP), a dairy effluent obtained from whey valorization processes. Although attractive fermentation performances were reached, significant improvements are required to eliminate recombinant plasmids, antibiotic resistances and inducible promoters, and increase ethanol tolerance. Here, we report a new strain with chromosomally integrated ethanologenic pathway under the control of a constitutive promoter, without recombinant plasmids and resistance genes. The strain showed extreme stability in 1-month subculturing, with CWP fermentation performances similar to the ethanologenic plasmid-bearing strain. We then investigated conditions enabling efficient ethanol production and sugar consumption by changing inoculum size and CWP concentration, revealing toxicity- and nutritional-related bottlenecks. The joint increase of ethanol tolerance, via adaptive evolution, and supplementation of small ammonium sulphate amounts (0.05% w/v) enabled a fermentation boost with 6.6% v/v ethanol titer, 1.2 g/L/h rate, 82.5% yield, and cell viability increased by three orders of magnitude. Our strain has attractive features for industrial settings and represents a relevant improvement in the existing ethanol production biotechnologies.

**Supplementary Information:**

The online version contains supplementary material available at 10.1186/s13068-023-02298-z.

## Introduction

The valorization of abundant waste materials to produce valuable compounds requires high-efficiency and cost-effective processes for addressing circular economy requirements in several sectors, such as biofuels and renewable energy. Whey is an abundant dairy wastewater generated from cheese manufacturing [1]. Despite whey having been used as animal feed or discharged in river systems or municipal sewage for a long time [2, 3], it is currently classified as a special waste in many countries due to its high biochemical and chemical oxygen demand [4, 5]. For this reason, disposal of whey leads to waste management issues and represents a limitation in the development of small to medium dairy industries [6]. Thanks to the high nutritional content of whey, extraction processes of different compounds such as lipids and proteins for use as food additives, therapeutics, energy drinks, cosmetics and nutraceuticals have been designed [6–8].

Whey permeate (WP) is an abundant lactose-rich waste liquid derived from the cream and protein extraction process by skimming and ultrafiltration of whey, respectively, and is also a special waste mainly because of its high lactose content [3]. WP is usually processed via reverse osmosis to reduce its volume for transportation, thus obtaining concentrated WP (CWP). Several technologies were proposed to further valorize WP and CWP, exploiting their high lactose content to produce prebiotic oligosaccharides, carotenoids, oil, lactic acid, nanoparticles, biogas, biohydrogen and polyhydroxyalkanoate [3, 6, 9–13]. WP and CWP were also proposed as co-substrate additives for other fermentation processes [14, 15].

One promising technology is the use of natural or engineered microbial biocatalysts, such as bacteria [16–18] or yeasts [19], to ferment lactose into ethanol fuel. Although advances in strain performances have been proposed and examples of industrial implementation have also been reported, the dairy waste-to-ethanol conversion technology still needs improvements to guarantee its wide adoption, based on the requirements of different bioeconomic models [15, 20, 21].

Motivated by this environmental and bioenergy challenge, we previously constructed an engineered ethanologenic strain using the lactose-consuming non-pathogenic *Escherichia coli* W chassis by incorporating a synthetic ethanologenic pathway (including the codon-optimized *adhB* and *pdc* genes, encoding the *Zymomonas mobilis* alcohol dehydrogenase II and pyruvate decarboxylase enzymes) [17], and then by deleting three competing pathways (fumarate reductase, lactate dehydrogenase and pyruvate formate lyase routes) [22]. The obtained metabolically optimized strain was able to efficiently grow and consume the lactose contained in WP and CWP at different dry matter levels (5–15% w/v) to produce ethanol as the main fermentation product. However, the developed strains still have drawbacks that limit their use in industrial settings, i.e., the presence of recombinant plasmids, antibiotic resistance genes (ARGs) and inducible promoters. These features are convenient for engineered strains prototyping, but are undesired in large-scale contexts [23]. In fact, plasmids in microbial populations generally suffer from structural and segregational instability due to DNA mutations in the target genes and loss during cell division, respectively [24, 25]. Both issues generate cells without a functional genetic program and, consequently, a decrease of productivity in industrial processes. Instability can be enhanced by the burden imposed by plasmid replication or the expression of additional plasmid-borne genes needed for its maintenance, e.g., replication genes or selection markers [26]. The occurrence of these drawbacks may depend on the specific plasmid, recombinant genes, media and growth conditions [26, 27]. In addition, the costs of antibiotics and chemical inducers, used to maintain plasmids and trigger the expression of the target genes, respectively, are often prohibitive in large-scale settings [28, 29]. The use of antibiotics in small- to large-scale contexts and their possible introduction in the environment also represents a global health threat, since it supports the development of antibiotic-resistant microbes, and leads to regulatory and wastewater management issues [26, 30]. The ARG itself used as a selection marker also has a crucial relevance for global health because of the potential horizontal gene transfer (HGT) of ARGs to other microbes. HGT can occur upon accidental release of recombinant strain or DNA in the environment, leading to the acquisition of novel resistance traits by pathogens [30]. Even in absence of mobilizable plasmids, ARGs can be horizontally transferred by transduction or natural transformation [31, 32]. Finally, plasmid copy numbers generally show a high cell-to-cell variability, with standard deviations comparable with their average values in the population; this feature may result in microbial sub-populations in low-producing state with a consequent performance drop of the process [33, 34].

The removal of all these plasmid-linked traits and the chromosomal integration of the ethanologenic pathway will provide a more stable genetic program with limited potential spreading of hazardous DNA, and no need for antibiotic selection or gene expression induction.

Ethanologenic strains with chromosomally integrated *pdc-adhB* operon were previously constructed [35–39]. However, none of them were optimized for lactose-rich waste fermentation and efficient ethanol production required complex nutrients (e.g., yeast extract or ammonium), osmoprotectants (e.g., betaine) and high expression of the operon either sustained by strong promoters or using tandem DNA copies of the genes of interest [16, 27, 36, 38, 40, 41]. All of these manipulations could lead to genetic instability due to transcriptional burden pressure or DNA rearrangements [42] and previous studies confirmed the limited stability of some of these strains during serial transfers [43, 44]. Instability is a relevant issue since a loss of ethanol production capability can result in a performance drop in continuous cultures or continuously propagated batch cultures; mitigation of this issue requires the preparation of fresh bacterial biomass for the inoculation of a reliable strain, consequently increasing fermentation steps and costs. Examples of chromosomally integrated chassis are the *E. coli* W, Crooks and BW25113 strains, engineered with the *pdc-adhB* operon under the control of a strong promoter such as P_G25_, P_LlacO1_, or the native promoter of the *rrnB* and *pflB* loci, in single or tandem copies. Although no fine tuning of transcription was carried out in these works, a decrease in promoter strength was reported to be detrimental to ethanologenicity [35, 39].

Conversely, our *adhB-pdc* operon, optimized in terms of codon composition and ribosome binding sites (RBSs), required very low transcription levels for ethanol production on a low-copy plasmid. This feature made our synthetic operon an excellent candidate for chromosomal integration to obtain a stable strain with no need for strong promoters or gene duplications.

Moreover, to support the definition of an efficient CWP fermentation bioprocess, variables such as nutritional requirements and tolerance to growth/fermentation inhibitors are worth investigating in the specific strain developed and wastewater used. In fact, although some of the strains described above have already been tested in lactose-containing media and optimal values were reproducibly found for some parameters (e.g., pH and oxygen), heterogeneous requirements for media supplements were observed in different engineered strains [16, 17, 22, 36, 40, 45–47].

In this work, we constructed a new bacterial strain with a chromosomally integrated ethanologenic pathway, and investigated its robustness and fermentation performance in CWP at different dry matter levels; finally, we evaluated the genetic and bioprocess features leading to further improvement in ethanol production. The final strain obtained in this work reached unprecedented ethanologenic performances for a bacterial biocatalyst and represents a promising technology for biofuel production from concentrated dairy waste.

## Materials and methods

### Strains and media

W_ΔLFP_ [22], a derivative of *E. coli* W (DSM 1116) [48], was used as the parent strain for construction of the new strains reported in Table 1. In our previous works, *E. coli* W was selected among different strains as an optimal host for dairy waste fermentation [17], and W_ΔLFP_ was constructed and characterized as an improved strain for ethanol production due to its low organic acid formation [22]. The W_ΔLFP_-pL13 strain, bearing the pL13 ethanologenic plasmid under the control of the *P*_lux_ promoter, was used in control experiments [17]. The W105F strain, bearing a constitutive ethanologenic operon in the *frd* locus, was selected among the integrated strains and used in most of the pH-controlled fermentation experiments. The W105Fe strain was derived from W105F by adaptive evolution, as detailed below.

**Table 1:** Strains used in this work

Strain	Genotype	References
W_ΔLFP_	W Δ*ldhA* Δf*rdAB* Δ*pflB-focA*	[22]
W_ΔLFP_-pL13	W_ΔLFP_ transformed with ethanologenic plasmid pL13^a^	[22]
W117L	W_ΔLFP_ *ldhA*::*P*_117_-*adhB-pdc*^a^	This study
WluxL	W_ΔLFP_ *ldhA*::*P*_lux_-*adhB-pdc*^a^	This study
WluxF	W_ΔLFP_ *frdAB*::*P*_lux_-*adhB-pdc*^a^	This study
W105F	W_ΔLFP_ *frdAB*::*P*_J105_-*adhB-pdc*^a^	This study
W105Fe	W105F after adaptive evolution to improve ethanol tolerance	This study

^a^DNA sequences are available in the Registry of Standard Biological Parts (http://parts.igem.org) with the following accession numbers. *P*_J105_ promoter: BBa_J23105; *P*_J117_ promoter: BBa_J23117; *P*_lux_ promoter: BBa_R0062; promoter-less *adhB-pdc* synthetic operon with terminator and strong RBSs: BBa_K173020; pL13 plasmid: pSB4C5 vector backbone with BBa_K173022 as insert

L-broth (LB; 1% NaCl, 1% tryptone, 0.5% yeast extract; 1.5% agar for plates) was used to grow bacteria for cloning. LB with 4% lactose (LBlac) was used to prepare inoculum for fermentation experiments. MacConkey agar (#01-118, Scharlab) was adopted to screen for ethanologenic strains by a colorimetric plate assay on colonies producing low amounts of acids during lactose fermentation. Chloramphenicol at 12.5 μg/mL was used to propagate strains with the pL13 plasmid, unless differently indicated. CWP was retrieved from the Cazzago San Martino whey valorization plant (Serum Italia S.p.A., Italy). It was typically characterized by a 15% dry matter, pH 6.0, protein content of 6 g/L, sugar content of 120 g/L, of which > 98% was lactose and the remaining part included galactose and/or glucose. CWP, routinely stored at − 20 °C, was thawed and filtered using a custom dead-end filtration system with DKF20 sheets (Filterflo s.r.l., Binasco, Italy) as previously described [22]. CWP_1:2_, CWP_2:3_ and CWP_3:4_ were obtained by 1:2, 2:3 and 3:4 dilutions, respectively, of CWP with sterile deionized water for fermentation tests with lower dry matter. Another batch of CWP, referred to as CWP_R_, was obtained previously from the Recetto whey processing plant (Negri Alimenti S.p.A., Italy) and was used for comparisons as undiluted medium or as a twofold dilution (CWP_R1:2_) [22]. When specified, 150 mM piperazine-*N*,*N*′-bis(2-ethanesulfonic acid) (PIPES, pH 7.0) was added to CWP to buffer pH drop in test tube fermentations [22], while ammonium sulphate (0, 0.005, 0.02, 0.05, or 0.2% w/v) was added as a nutritional supplement.

### Strain construction

Four strains (W117L, WluxL, WluxF and W105F) were newly constructed by chromosomally integrating a constitutively expressed and optimized *adhB-pdc* operon in W_ΔLFP_, varying the promoter (*P*_J117_, *P*_J105_ or *P*_lux_, without the *luxR* activator gene [49]) and the integration locus (*ldhA* or *frdA*). BioBrick Standard Assembly [50] was used to construct integrative plasmids based on the pBBknock genetic tool [51]. Integrative plasmids included two regions flanking the *adhB-pdc* operon, homologous to the flanking regions of the target locus. The homologous regions for *ldhA* and *frdA* were used previously to disrupt the two competing pathways [22, 51] and in this work they were adopted for operon insertion. The pBBknock integration procedure was used and the resulting catechol-negative colonies were screened by PCR, DNA sequencing, chloramphenicol sensitivity and low acid production on McConkey plates. Restriction digestions, DNA extraction from bacterial cultures or 1% agarose gel electrophoresis, ligation and plasmid extraction from bacteria were performed via standard protocols by Thermo Fisher enzymes and Macherey–Nagel DNA extraction kits, following the manufacturer instructions. Bacterial transformation was performed via heat-shock at 42 °C. Long-term stocks were prepared by mixing glycerol with saturated cultures grown in LB, to reach a 20% glycerol concentration, and stored at − 80 °C.

### Evolutionary stability characterization

Strains from long-term stocks were streaked on LB agar and incubated overnight (16–24 h) at 37 °C. For each strain, single colonies were used to inoculate three 15-mL tubes containing 2 mL of LBlac. Cultures were incubated at 37 °C, 220 rpm overnight. Saturated cultures were 100-fold diluted in 5 mL of CWP_1:2_ + PIPES in 50-mL tubes, which were incubated under the same conditions as above for 30 days. Every 5 days, strains were subcultured by 100-fold dilutions in fresh CWP and a properly diluted aliquot was plated on MacConkey agar. Plates were incubated overnight at 37 °C and the percentage of mutants in the lactose-to-ethanol pathway was calculated as the number of purple colonies divided by the total number of colony forming units (CFUs). Typically, around 200 CFUs were counted in each plate.

### Adaptive laboratory evolution

Single colonies from a streaked MacConkey plate were used to inoculate 5 mL of CWP_1:2_ supplemented with ethanol at progressively increasing concentrations (1.5, 2, 2.5, 3.5, 4 and 4.5% w/v) in 50-mL tubes. Cultures were incubated at 37 °C, 220 rpm for 72 h. Serial dilutions of bacteria were plated on MacConkey agar and incubated at 37 °C overnight. At each step, the procedure above was repeated in parallel for 5–7 colonies until the target tolerance of 4.5% w/v was reached (about 1.5 months), representing an about threefold improvement in the tolerance compared with the parent strain. A single clone was selected among the evolved ones that survived at the highest ethanol concentration and long-term stored at − 80 °C as W105Fe. The other ethanol-tolerant clones obtained, stored as a backup strategy, were not further analyzed since W105Fe showed satisfactory ethanol production and tolerance performances, and was used in the downstream tests.

### Fermentation experiments

Fermentation tests were performed on laboratory scale in test tubes or bioreactor at volumes of 5 mL or 600 mL, respectively. A large headspace volume was adopted (90% in test tubes, 78% in bioreactor) to maximize ethanol production performance, based on previous data on our strain and others [22, 47].

For test tube experiments, single colonies from a streaked LB agar plate were used to inoculate 1.2 mL of LBlac in 15-mL tubes, which were incubated overnight at 37 °C, 220 rpm. Cultures were centrifuged (10 min, 4000 rpm), the supernatant was discarded and the pellet was resuspended in 5 mL of CWP + PIPES in 50-mL tubes at the indicated dilution. Cultures were incubated under the same conditions as above for 72 h.

The inocula for pH-controlled bioreactor experiments were prepared as above in a proper volume of LBlac. Saturated cultures were used to inoculate 600 mL of CWP media, at different inoculant concentrations, i.e., 1:5, 1:10, 1:50 or 1:500 ratios between saturated culture and CWP volume. For the 1:5 and 1:10 inoculum conditions, the culture grown in LBlac was centrifuged (10 min, 4000 rpm), the supernatant was discarded and the pellet was used for the inoculum to avoid high carry-over of spent LBlac. The choice of a 1:5 inoculum ratio as the standard fermentation condition for test tubes and pH-controlled experiments was based on our previous works [17, 22], in which the same parameter value was used and performance comparisons were therefore facilitated. The 600-mL culture was incubated in a glass thermo-jacketed 3-L vessel (Applikon) for 96 h, unless differently indicated. Temperature, pH and agitation were maintained at 37 °C, 6.5 and 300 rpm, respectively. KOH 3 M was used for pH control via a Masterflex Easy Load pump head.

In all fermentation experiments, culture samples were taken at specific time points, filter-sterilized (0.2 μm) and stored at − 20 °C for further HPLC analysis. Samples were also diluted and plated on LB agar for CFU analysis after incubation at 37 °C.

### Analysis of fermentation products

An LC-2000 HPLC system (Jasco) equipped with an RID 10A detector (Shimadzu) and an autosampler was used to quantify sugars (lactose, glucose, galactose), ethanol and fermentation by-products (lactic, succinic and acetic acid). A Supelco C-610H 30 cm × 7.8 mm column (59320-U, Sigma Aldrich) was used to measure sugars, ethanol and acetic acid, using 0.1% H3PO4 as mobile phase, 0.5 mL/min flow rate, 30 °C and 25 μL sample injection. An AQUASIL C18, 5 μm, 25 cm × 4.6 mm (Thermo Scientific) was used to measure succinic acid, using 50 mM KH_2_PO_4_ at pH 2.8 as mobile phase, 1.25 mL/min flow rate, 25 °C and 10 μL sample injection. A Repromer H, 9 μm, 25 cm × 8 mm (Dr Maisch GmbH, Ammerbuch-Entringen, Germany) was used to measure lactic acid, using 1 mM H_2_SO_4_ as mobile phase, 0.5 mL/min flow rate, 50 °C and 10 μL sample injection. The ChromNAV 2.0 software (Jasco) was used to analyze chromatograms.

### Analysis of fermentation performance

Ethanol titer, production rate and conversion yield were used as the main performance indicators. Titer was recorded as the ethanol concentration at the end of experiments. A correction for the injected volumes of KOH was applied in tables and pie charts [22], as indicated, but not in fermentation profile graphs. Production rate was computed as the highest numeric time derivative value of the ethanol concentration profile. Conversion yield for ethanol and by-products was calculated as a fraction of the theoretical conversion yield, which was computed considering the reaction stoichiometry of 4 molecules of ethanol and organic acids per molecule of lactose, and 2 molecules of glucose and galactose per molecule of lactose. Although lactose was the main sugar in CWP, glucose and galactose (initially accounting for < 2% of total sugars) could be increasingly detected over time, probably due to lactose hydrolysis by β-galactosidase from dead bacteria. For this reason, the analyses included residual sugars as the sum of lactose, glucose and galactose, instead of lactose alone. Metabolic profiles in pie charts were used to present sugar and fermentation product distribution. The measured concentration of lactic acid, which is initially present in CWP and is also consumed over time, was corrected by subtracting the minimum measured concentration in the time series [22].

Two-sided unpaired t-tests, performed via Microsoft Excel, were used to compare the average performance indexes between two strains or conditions, measured in duplicate experiments. The *corrcoef* Matlab R2017b (MathWorks, Natick, USA) function was used to compute correlations (*ρ*) and their *p*-values (*p*). Correlation analyses were carried out on performance indexes of the same strain under varying conditions (*N* ≥ 3) of CWP dilution or inoculum size, each of them measured in single- or two-replicate experiments, as indicated. Statistical significance was considered with values of *p* < 0.05. Graphs were generated using GraphPad Prism 8.3.0.

## Results and discussion

### Design and construction of a stable ethanologenic strain

**Fig. 1:**
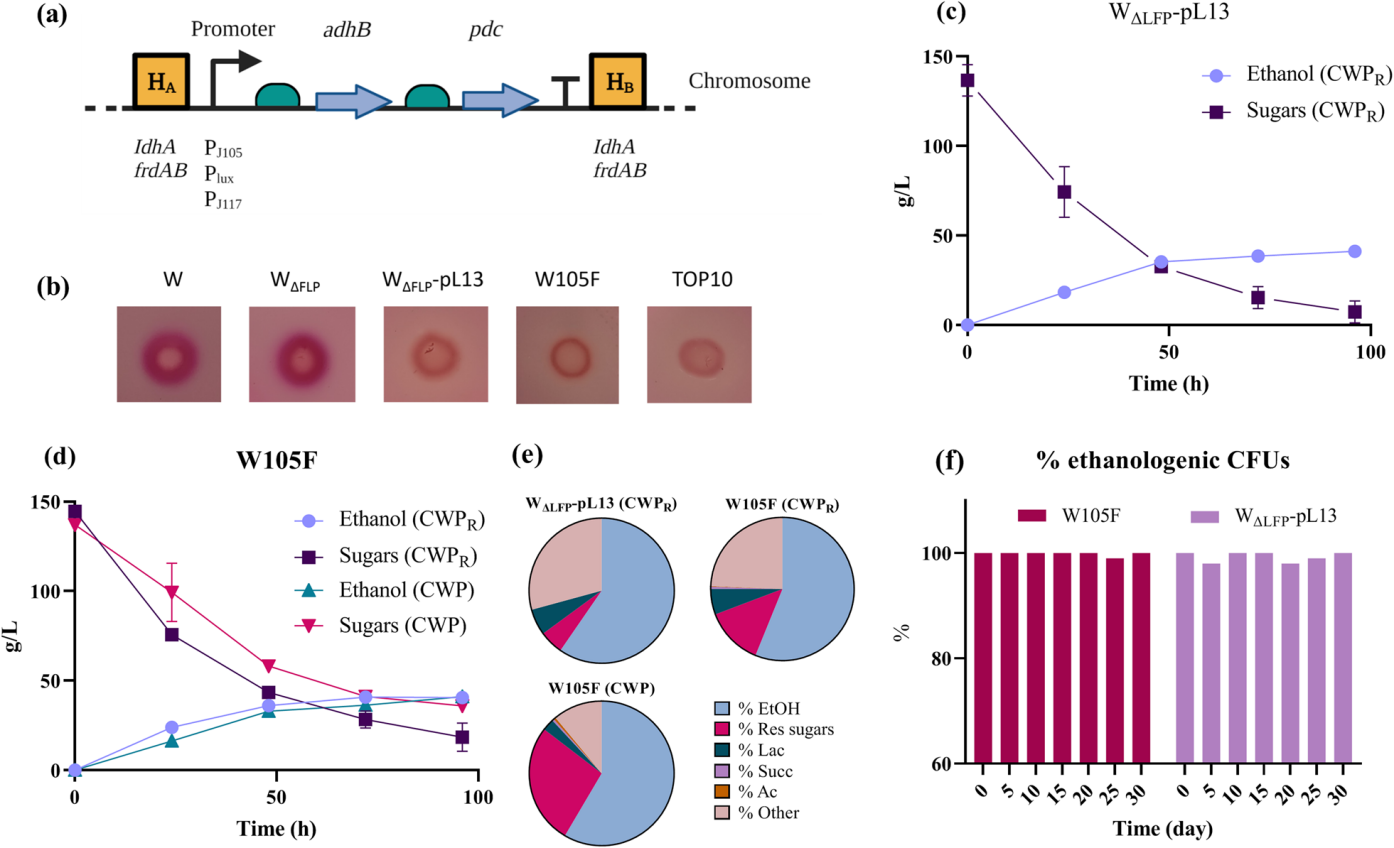
Description and fermentation performance of the W105F strain in CWP_R_ and CWP. **a** Design of a chromosomally integrated ethanol production pathway, indicating the candidate promoters and the integration loci. Straight arrows: coding sequences; curved arrows: promoters; half ovals: ribosome binding sites (RBSs); T symbol: transcriptional terminator; straight line: chromosomal DNA. The H_A_ and H_B_ regions represent the DNA sequences flanking the integration site that are used in homologous recombination, with the two selected loci in which integration occurred listed below. **b** MacConkey plate qualitatively showing the different color outputs of the indicated strains. For each strain, 2 μL of culture from long-term stock was pipetted on a non-selective MacConkey plate and incubated overnight at 37 °C. Control spots were prepared by using the W strain (DSM 1116), which has a wild-type mixed acid fermentation, and the TOP10 *E. coli* strain (Invitrogen), which is unable to consume lactose and no mixed acid fermentation is expected to occur. **c, d** Fermentation time course data from pH-controlled bioreactor experiments. Time series indicate the concentrations of sugars and ethanol with data points representing the average of two replicates and error bars the standard deviation. **e** Metabolic profiles from pH-controlled bioreactor experiments. Pie charts represent the distribution of residual sugars (*Res sugars*), fermentation products (*EtOH*, ethanol; *Lac*, lactic acid; *Succ*, succinic acid; *Ac*, acetic acid) and non-recovered carbon (*Other*). The **c**–**e** panels correspond to data of the plasmid-based ethanologenic W_ΔLFP_-pL13 strain, used as a reference, in CWP_R_ (**c**), the W105F strain in CWP_R_ and CWP (**d**) and the metabolic profiles in these conditions (**e**). **f** Time series indicating the percentage of W_ΔLFP_-pL13 or W105F bacterial population that maintained an ethanologenic phenotype over a month upon subculturing every 5 days

We rationally designed a set of candidate ethanologenic strains with chromosomally integrated *adhB-pdc* synthetic operon, without any recombinant plasmid or antibiotic resistance gene (Fig. 1a). We considered the performance of the plasmid-based W_ΔLFP_-pL13 strain as the maximum achievable target. W_ΔLFP_-pL13 strain had ~ 5 copies of the ethanologenic operon, maintained via a pSC101 replication origin, and a low transcription level guaranteed by the *P*_lux_ promoter in the off-state, i.e., without addition of the N-3-oxohexanoyl-l-homoserine lactone (HSL) inducer. In principle, a chromosomal integration in a single locus would result in a ~ 5 fold decrease in DNA copy number, which should be compensated by a fivefold increase in transcription using a stronger promoter to maintain the same enzyme levels. We assumed that the transcription level of our optimized *adhB-pdc* operon could also be lowered without affecting the ethanologenicity of the strain. In fact, the *P*_lux_ promoter has a very weak transcriptional activity in the absence of HSL, and an increase in the HSL concentration did not improve ethanol production, with detrimental effects on cell growth at high inducer levels [17, 22]. Therefore, we selected weak promoters with a 0.9- to 3.5-fold activity compared to *P*_lux_ (*P*_J117_ < *P*_lux_ < *P*_J105_). To explore possible position-dependent effects, two genomic loci were selected as integration sites (*ldhA* and *frdAB*), in which the parent strain had deletions in the two original loci, and we tested four of the promoter/locus combinations.

We conceived a convenient MacConkey plate assay to screen strains with chromosomally integrated ethanol pathway, by exploiting their lower acid production compared with non-ethanologenic ones, such as the W_ΔLFP_ parental strain. Even though the main routes for organic acid production were disrupted in W_ΔLFP_, we previously detected acids in fermentation experiments, most probably generated by alternative pathways [22]. Ethanologenic strains were characterized by yellow halos, while negative clones were surrounded by a purple area on agar plates (Fig. 1b).

An ethanol production screening in 72-h test tubes confirmed that all the four new strains were able to convert lactose from CWP_R1:2_ + PIPES into ethanol, as the main fermentation product (Additional file 1: Fig. S1). Data showed no relevant difference in ethanol titer between the two integration loci (see ethanol concentration in WluxL vs. WluxF, Additional file 1: Fig. S1). The W105F strain was selected among the four candidates, due to its superior lactose consumption, ethanol production and cell density. In this experimental condition, none of the strains reached the ethanol titer and lactose consumption performance of the plasmid-based W_ΔLFP_-pL13 strain. However, 72-h pH-controlled bioreactor experiments showed that W105F and W_ΔLFP_-pL13 had a comparable ethanol titer (43.7 vs. 43.8 g/L), production rate (1 vs. 0.82 g/L/h), conversion yield (62% vs. 63%), cell viability profile and distribution of fermentation products (less than twofold differences) in CWP_R_ fermentations (Fig. 1c, d, Table 2 and Additional file 1: Fig. S2A). KOH consumption and residual sugars were slightly higher for W105F than for W_ΔLFP_-pL13, but values remained within reasonable ranges and with no statistical difference between the two strains. To our knowledge, this is the first stable bacterial strain able to maintain high ethanologenic performance, comparable with those of plasmid-based solutions, and performing in the absence of any media supplementation through the activity of a weak promoter for the *adhB-pdc* operon.

**Table 2:** Fermentation performance indicators in pH-controlled bioreactor experiments

Strain	Medium	Inoculum ratio	Ethanol titer (g/L)^a^	Maximum production rate (g/L/h)	Yield (%)^a^	KOH (mmol/L)	Residual sugars (%)^a^
W_ΔLFP_-pL13^b^	CWP_R_	1:5	43.77 (1.2)^c^	0.82 (0.01)	62.97 (0.49)	192.5 (45.96)	5.6 (4.46)
W105F	CWP_R_	1:5	43.69 (1.04)	1 (0.02)	61.96 (0.39)	232.5 (45.96)	13.63 (5.49)
W105F	CWP	1:5	43.07 (0.14)	0.75 (0)	80.05 (1.99)	142.5 (24.75)	27.45 (1.43)
W105F	CWP_3:4_	1:5	37.19	0.67	70.95	110	13.5
W105F	CWP_2:3_	1:5	34.24 (2.33)	0.68 (0.16)	74.8 (6.22)	170 (28.28)	4.33 (3.66)
W105F	CWP_1:2_	1:5	31.51	0.65	88.95	150	0
W105F	CWP_2:3_	1:10	28.82	0.45	86.67	110	30.44
W105F	CWP_2:3_	1:50	19.9 (0.64)	0.31 (0.08)	74.54 (3.42)	140 (0)	46.36 (5.35)
W105F^d^	CWP + (NH_4_)_2_SO_4_^e^	1:5	45.83 (0.99)	0.91 (0.13)	97.33 (30.04)	157.5 (95.46)	31.08 (15.69)
W105Fe	CWP	1:5	37	0.79	87.3	125	32.65
W105Fe^d^	CWP + (NH_4_)_2_SO_4_	1:5	54.11 (0.95)	1.2 (0.14)	82.54 (3.3)	125 (21.21)	12.8 (10.79)
W105Fe	CWP + (NH_4_)_2_SO_4_	1:50	54.84	1.06	95.68	170	29.7
W105Fe	CWP + (NH_4_)_2_SO_4_	1:500	35.59	1.43	79.31	130	45.96

^a^Corrected by considering the volume of dispensed KOH to adjust pH, measured at the end of the experiments

^b^Chloramphenicol was added to the fermentation medium

^c^Numbers in brackets represent standard deviations, for the experiments with replicates

^d^In one of the two replicates, the experiment was stopped at 72 h instead of 96 h

^e^Ammonium sulphate concentration is 0.05% w/v

After the closure of the Recetto plant, we switched from CWP_R_ to CWP from the Cazzago San Martino plant as a fermentation medium for all the subsequent experiments. Fermentations in pH-controlled bioreactors showed that, in the new CWP, W105F could reach a similar ethanol titer (43 g/L) and a slightly lower production rate (0.75 g/L/h). However, the metabolic profile of the strain was largely different when grown in CWP_R_ and CWP (Fig. 1e). A twofold higher percentage in residual sugars was observed in CWP with respect to CWP_R_ (27% vs. 13%, *p* < 0.05). Also, lactic acid percentage was twofold lower in CWP with respect to CWP_R_, and a much higher carbon recovery was obtained in CWP (89% vs. 76%). As a result, the ethanol conversion yield was also higher in CWP than in CWP_R_ (80% vs. 62%) despite not being statistically different due to the large variation of this index and the low number of replicates performed (*N* = 2). Nonetheless, data clearly demonstrate that both wastewaters can be used for ethanol production even though their fermentation profiles show relevant variations. Such differences were surprising, since whey permeate was expected to show low variability among different industrial plants due to the relatively standardized source material (mixture of whey collected from different cheese manufacturing processes) and processing steps (ultrafiltration, reverse osmosis). In our case, sugars, acids and dry matter levels were comparable between CWP and CWP_R_. In this work, CWP_R_ was not further considered, but this evidence raises warnings on the generalization of valorization processes using CWP from different sources without a proper characterization.

The MacConkey assay was finally adopted to quantify the loss of ethanol production phenotype upon serial transfers of W105F for 1 month. Results highlighted an excellent stability, since nearly all the colonies on plates showed ethanologenic phenotype (Fig. 1f). The control strain (W_ΔLFP_-pL13), assayed without antibiotic selection, also showed nearly 100% stability, confirming previous results on a pSC101-based vector which showed no detectable loss for 150 generations, using a different strain and growth medium [52]. Our data showed that the W105F strain is characterized by a much higher evolutionary stability than the chromosomally integrated ethanologenic strains in previous studies, for which a loss of ethanologenicity was observed after less than a month [43, 44, 53]. Even though our chromosomally integrated and plasmid-based solutions exhibited comparable evolutionary stability in absence of antibiotics and inducers, the new W105F strain has key benefits over W_ΔLFP_-pL13, primarily because the potentially hazardous sequences (i.e., the chloramphenicol resistance gene) have been removed. Other unnecessary sequences (i.e., the *luxR* expression cassette and the pSC101 replication origin) and repetitive DNA (e.g., a second transcriptional termination sequence downstream of *luxR*), included in pL13, are also not present in W105F, reducing the size of the genetic program from 7.3 to 3.1 Kbp. Finally, since plasmid burden is known to be function of media and growth conditions [27, 44], pL13-bearing cultures may still show instability and/or significant fractions of non-producing bacteria in other experimental conditions not tested in this work (e.g., another wastewater or in different culture modes), making our integrated strain a more promising option for ethanol production in future applications.

### Tuning of waste and strain concentrations

To investigate conditions leading to efficient sugar consumption and ethanol production in W105F, different CWP concentrations and inoculum sizes were tested. Some of these experiments were run with a single replicate to explore possible trends in fermentation indexes to find fermentation bottlenecks.

**Fig. 2:**
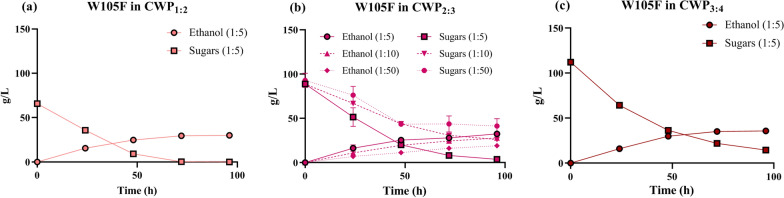
Fermentation performance of the W105F strain in CWP with different dilutions and different inoculum sizes. Fermentation time course data from pH-controlled bioreactor experiments are represented as concentration time series of sugars and ethanol. Data points represent concentrations of a single replicate or the average of two replicates (as reported in Table 2), with error bars corresponding to the standard deviation. Panels correspond to fermentations in CWP with different dilutions: CWP_1:2_ (**a**), CWP_2:3_ (**b**) and CWP_3:4_ (**c**) for the standard bacterial inoculum size (1:5 ratio). For the CWP_2:3_ condition, data are shown for the standard inoculum size and for the 1:10 and 1:50 ratios (**b**). The number in brackets in the legend indicates the inoculum size

As expected, a decrease in the CWP concentration resulted in changes in ethanol titer, from 31.5 to 43 g/L, and in residual sugars, from 0 to 27.5% (Table 2 and Fig. 2a–c). Both indexes showed a high and significant positive correlation with the CWP concentration (*ρ* = 0.984 and 0.977, respectively—*p* < 0.05), consistent with previous observation in CWP_R_ at different dry matter values (8–15%) [22]. Ethanol production rate also varied from 0.65 to 0.75 g/L/h and showed a high positive correlation with initial sugars (*ρ* = 0.984), as expected from previous tests with CWP_R_ [22], though not statistically significant and with values only varying by 14% among the CWP concentrations. Conversely, ethanol conversion yield and KOH consumption did not show clear trends, even though data confirmed that a higher yield than the one obtained with CWP_R_ was systematically achieved (> 71% vs. 62%, see Table 2). The 2:3 dilution was selected for further testing since it enabled a > 95% consumption of the initial sugars with reasonable ethanol titer (34.2 g/L), rate (0.68 g/L/h) and yield (75%).

We then carried out CWP_2:3_ fermentations with 2- and 10-fold lower amounts of inoculum, corresponding to 1:10 and 1:50 volume ratios between saturated culture and CWP_2:3_. The standard inoculum condition in CWP_2:3_ corresponded to a 1:5 volume ratio and was used for comparison. A decrease in bacterial inoculum resulted in a dramatic drop in CWP_2:3_ fermentation indexes, demonstrated by the high positive correlation of inoculum size with ethanol titer (*ρ* = 0.979, *p* > 0.05) and rate (*ρ* = 0.998, *p* < 0.05), both showing an about twofold variation range, and by the high negative correlation of inoculum size with residual sugars (*ρ* = − 0.997, *p* < 0.05), showing an about 11-fold range (Table 2 and Fig. 2b). An inoculum reduction by only twofold already resulted in lower titer and rate, and higher residual sugars. The tenfold inoculum reduction resulted in unattractive performance indexes: 20 g/L ethanol titer, 0.31 g/L/h production rate and 46% residual sugars. As before, conversion yield did not show clear inoculum-dependent trends.

Altogether, data suggest the presence of factors limiting ethanol production at high lactose concentrations. Toxicity due to high ethanol levels may be responsible for the incomplete sugar consumption observed in CWP and diluted CWP. Nutrient limitation could also contribute to the performance drop when a decreasing amount of bacterial biomass is inoculated. Both assumptions are consistent with the cell viability drop observed previously [22] and in this work in all the fermentation experiments, even in the tests with low ethanol titer (Additional file 1: Fig. S2B). The two factors were therefore tested, as described in the section below.

### Increasing ethanol tolerance and fermentation performance

**Fig. 3:**
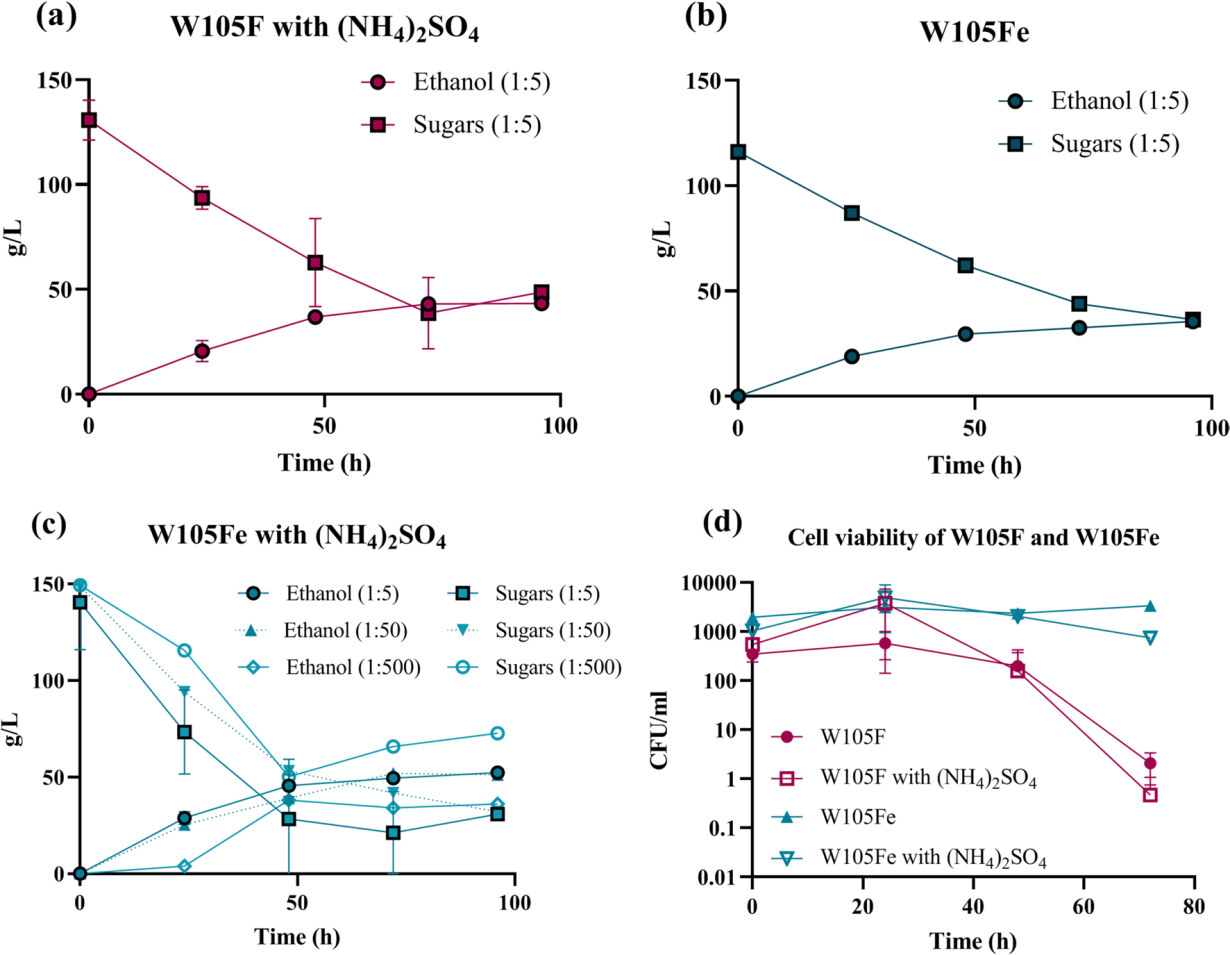
Fermentation performance of the W105F and W105Fe strains in CWP with different supplements and inoculum sizes. Fermentation time course data from pH-controlled bioreactor experiments are represented as concentration time series of sugars and ethanol, or CFU time series. Data points represent values from a single replicate or the average of two replicates (as reported in Table 2), with error bars corresponding to the standard deviation. Panels correspond to fermentations in CWP with different strains and conditions: sugars and ethanol for W105F in CWP + ammonium sulphate (**a**), sugars and ethanol for W105Fe in CWP (**b**), sugars and ethanol for W105Fe at the 1:50 and 1:500 inoculum ratios in CWP + ammonium sulphate (the standard 1:5 inoculum ratio is shown for comparison) (**c**), cell viability of W105F and W105Fe in CWP and CWP + ammonium sulphate (**d**)

To support fermentation completion at high sugar concentrations, ethanol tolerance was addressed. Ethanol is known to be toxic for *E. coli* at concentrations above 1.5% w/v that may indeed limit production at late stages of fermentation [54]. Adaptive evolution has been reproducibly used previously to generate ethanol-tolerant strains [55, 56]. We followed a similar procedure starting with the W105F strain and sub-culturing it for more than 1 month in CWP_1:2_ at increasing ethanol concentrations, constantly checking the maintenance of the ethanologenic pathway by MacConkey plates. The strain was eventually able to grow in 4.5% w/v ethanol, not considering the ethanol additionally produced during culturing (not measured). Based on a test conducted at the end of the evolution process, the evolved strain, named W105Fe, maintained ethanol production features from CWP in pH-controlled bioreactor, although indexes were not higher than W105F and a slightly lower ethanol titer was recorded (Table 2 and Fig. 3b). As expected, W105Fe maintained a much higher cell viability than W105F in the same conditions even when the 3.5% w/v ethanol concentration was reached in the fermentation broth (Fig. 3d). The maintenance of high cell viability is an attractive feature for possible reuse of bacterial biomass.

To address possible nutrient limitation, ammonium sulphate was investigated as a supplement, since whey permeate is nitrogen-poor, and nitrogen addition was expected to improve carbon metabolism and therefore increase fermentation performance [6]. We first screened different ammonium sulphate concentrations in test tube experiments for the non-evolved W105F strain in CWP + PIPES. High ethanol production was observed at 72 h with 0.05% w/v ammonium sulphate (Additional file 1: Fig. S3). The same ammonium sulphate-dependent pattern was also observed for W105Fe (Additional file 1: Fig. S3). The value (> 40 g/L ethanol) reached with ammonium sulphate supplementation represents the highest titer obtained so far with engineered *E. coli* in dairy waste in test tubes, without strict pH control. Tests in pH-controlled bioreactor showed that W105F in CWP + 0.05% w/v ammonium sulphate had systematic but modest and not statistically significant improvements in titer (1.1-fold), rate (1.2-fold) and yield (1.2-fold) compared with the same strain without supplements (Fig. 3a and Additional file 1: Fig. S4A), suggesting that ammonium sulphate alone was insufficient to overcome fermentation bottlenecks. The maximum ethanol titer that was reached in pH-controlled fermentations without engineering ethanol tolerance was 45.8 g/L (corresponding to 43.5 g/L without correction by dispensed KOH) and cell viability drop was still observed (Fig. 3a, c), confirming that ethanol toxicity could have a negative impact on ethanol production by the non-evolved W105F strain.

However, the joint use of W105Fe and 0.05% w/v ammonium sulphate in bioreactor tests showed a great improvement in ethanol production, with 54.1 g/L titer (corresponding to 6.9% and 6.6% KOH-corrected and uncorrected v/v ethanol concentrations, respectively), 1.2 g/L/h rate and 82.5% yield (Fig. 3c and Additional file 1: Fig. S4B). To our knowledge, such indexes represent the highest performance achieved with an ethanologenic bacterial strain in dairy wastewater fermentation. Compared with the W105F strain in undiluted CWP without ammonium sulphate, significantly higher ethanol titer (1.25-fold, *p* < 0.05), production rate (1.6-fold higher, *p* < 0.05) and cell viability (1300-fold at 72 h, *p* < 0.05) were observed. Residual sugars (2.1-fold lower, *p* > 0.05) also showed a promising decrease. These data demonstrate that ethanol toxicity and nitrogen limitation play a crucial role, but only their simultaneous tackling was able to boost ethanologenic performance. Most probably, the synergistic impact of ethanol tolerance and nitrogen supplementation led to a more permissive growth environment in which ethanol toxicity was mitigated and fermentation could proceed for longer thanks to the improved carbon metabolism. Conversely, the effect of nitrogen supplementation alone was neutralized by ethanol toxicity, and ethanol tolerance alone led to a higher cell viability without improving ethanol production, probably due to suboptimal nitrogen availability in terms of low amount and limited assimilation of whey proteins [16, 57, 58].

In an attempt to further improve the sustainability of the process, we challenged W105Fe in CWP fermentations with ammonium sulphate at low inoculum concentration, a parameter that was previously shown to dramatically affect fermentation performance. Inocula at 1:50 and 1:500 volume ratios were tested, representing 10- and 100-fold lower bacterial amounts, respectively. In both inocula conditions similar and very high production rate and yield, comparable with the standard inoculum size, were obtained (Table 2 and Fig. 3c). As observed in the previous section, ethanol titer and residual sugars positively and negatively correlated with inoculum amount, respectively, suggesting that additional efforts are still needed to guarantee optimal bioconversion performance in conditions of low inoculum size, although correlation values were not statistically significant. Nonetheless, the ethanol titer was reasonably high in all the conditions and a much lower sensitivity to inoculum size (here tested with a 100-fold range) was observed than in the conditions with W105F without ammonium sulphate in CWP_2:3_, described in the previous section (tested with inoculum sizes in a tenfold range, already showing a dramatic performance drop); the experiment with a 1:50 inoculum ratio resulted in a 54.8 g/L ethanol titer, very similar to the attractive values obtained at the standard inoculum ratio of 1:5.

## Conclusions

In this work, we reported a novel bacterial biocatalyst that showed, to our knowledge, the highest ethanologenic performance obtained so far for an *E. coli* strain from dairy waste in terms of ethanol titer, rate and conversion yield.

As a major advantage compared with previous solutions, the reported strain has no recombinant plasmids, antibiotic resistance genes, inducible promoters, and has a low transcription level of the ethanologenic genes in a single chromosomal copy. Such features make this biocatalyst suitable for large-scale fermentations since no antibiotics and chemical inducers are required and there are no potentially unstable or hazardous genetic elements. The strain was demonstrated to stably maintain the lactose-to-ethanol conversion pathway for at least 1 month of continuous sub-culturing, largely surpassing the low evolutionary stability of previously reported strains with chromosomally integrated ethanologenic genes [43, 44, 53].

The characterization of our W105F strain was useful to drive metabolic and bioprocess interventions to overcome crucial bottlenecks that limited sugar consumption and ethanol production under standard and sub-optimal fermentation conditions, e.g., high sugar concentration or reduced inoculum size. When ethanol tolerance was increased (obtaining the W105Fe evolved strain) and ammonium sulphate (0.05% w/v) was added to CWP, we not only achieved the high performance mentioned above, but also limited the sensitivity to inoculum size, i.e., reasonable performances were obtained even for 10- to 100-fold lower bacterial biomass, and cell viability was increased by more than three orders of magnitude at 72 h, probably due to the improved ethanol tolerance.

The relevance of the results obtained is threefold. First, we demonstrated efficient fermentation of a highly concentrated lactose-rich waste stream, enabling sufficient ethanol titer to make ethanol extraction industrially attractive. Second, the significant cell viability increase herein obtained can support the reuse of the bacterial biomass for possible reinoculation. Third, although ammonium sulphate supplement corresponds to an additional cost to the process, its working concentration in this study to support ethanol production with engineered *E. coli* is fourfold lower than in previous reports [16, 40], thus foreseeing a potentially lower economic impact. In the tuning of all the process parameters such as fermentation time, media supplementation, inoculum size and initial CWP concentration, specific costs will have to be taken into account to drive future studies in pilot-scale plants. In fact, many of the parameters investigated in this work can also influence process costs in terms of supplement, energy, investments and waste disposal. For example, longer fermentation time or smaller inoculum size result in a decrease of bioreactors turnover and may require additional equipment to meet a target productivity; CWP dilutions resulting in a decrease in ethanol titer may decrease fermentation time and residual sugars, but will result in an increase in distillation energy per ethanol volume to be extracted; residual waste with a high organic load could represent a waste management issue and a relevant disposal cost. The quantification of such costs will be addressed in future studies by investigating CWP-to-ethanol production with fermentation and distillation technologies comparable with the ones of a large-scale industrial plant.

As a secondary product of this research, a convenient MacConkey plate assay was proposed to screen ethanologenic strains. Compared with the rosaniline bisulphite (RB) assay, previously proposed to screen strains with high alcohol dehydrogenase II activity [53, 59, 60], our method detects the functioning of the lactose-to-acetaldehyde bioconversion route, with the pyruvate-to-acetaldehyde being the last pathway step and the key metabolic branch between acids and ethanol production in our strain [17]. Our method includes a medium widely used in many laboratories, never used for this purpose, and adopts stable reagents and agar plates that can be routinely stored for weeks. Conversely, RB includes potentially hazardous and unstable reagents, and the long-term storage of plates is not recommended.

Overall, this work demonstrates that significant improvements can be designed and successfully implemented to the existing bacterial biocatalysts for ethanol production, and the resulting strain herein constructed represents a solution with highly attractive features towards sustainable industrial settings.

## Supplementary Information

**Additional file 1.** Supplementary figures.

## Data Availability

The data that support the findings of this study are available from the corresponding author upon reasonable request.
