# Supplementary material for: Design of a stable ethanologenic bacterial strain without heterologous plasmids and antibiotic resistance genes for efficient ethanol production from concentrated dairy waste
Source: Biotechnol Biofuels Bioprod. 2023 Apr 1;16:57. doi: 10.1186/s13068-023-02298-z (PMC10067303; doi:10.1186/s13068-023-02298-z)
Supplement: Supplementary file 1 — Additional file 1. Supplementary figures. [file 13068_2023_2298_MOESM1_ESM.pdf]

Design of a stable ethanologenic bacterial strain without heterologous plasmids and antibiotic resistance genes for efficient ethanol production from concentrated dairy waste

Supplementary figures

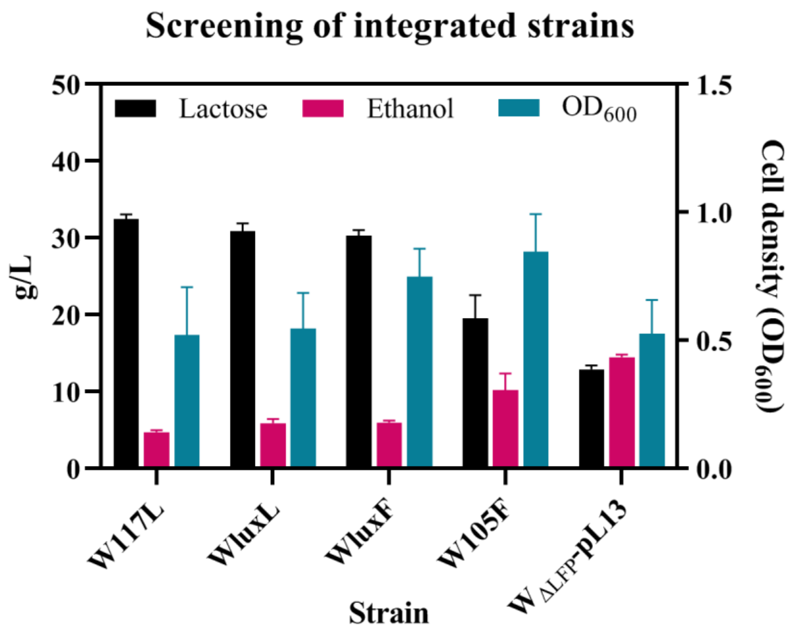

Figure S1. Fermentation performance screening in $CWP_{R1:2}$ + PIPES in test tube experiments for the four new ethanologenic strains constructed in this work with a chromosomally integrated *adhB-pdc* operon. Bars indicate the average lactose concentration, ethanol concentration and optical density at 600 nm (OD_{600}) after 72 h, for at least three replicates in the same day, with error bars corresponding to standard deviations. OD_{600} was used as a measure of cell density and was obtained by transferring 200 µl of properly diluted cultures into a 96-well plate and absorbance was acquired using an Infinite F200Pro plate reader (Tecan). Raw absorbance values were background-subtracted using $CWP_{R1:2}$ + PIPES and multiplied by their dilution factor to obtain the final OD_{600} value. Data for the plasmid-based $W_{\Delta LFP}$-pL13 strain, tested in parallel with the four candidate strains, are shown as a reference. Initial lactose concentration was 48.3 g/L.

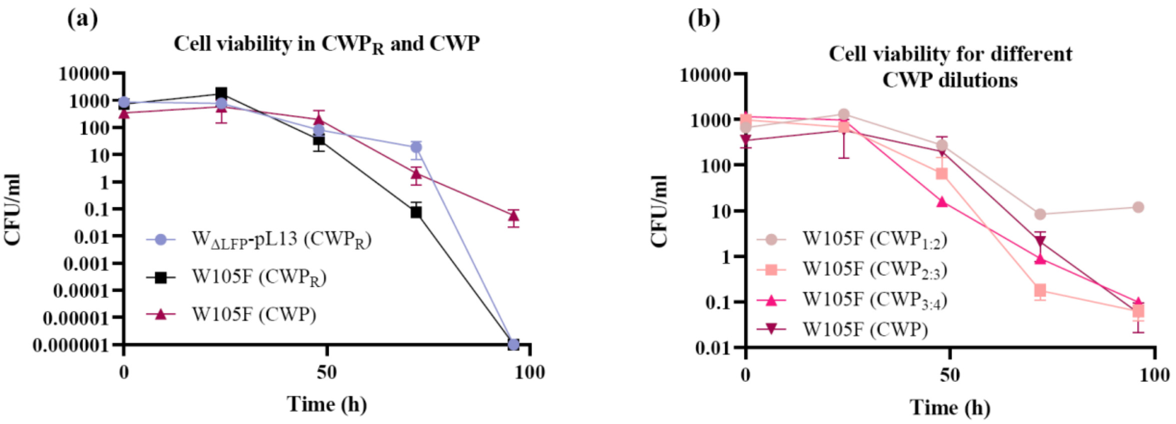

Figure S2. Cell viability time course for the W105F strain in different wastewaters, CWP_R or CWP (a) and in CWP at different dilutions (b) in terms of CFU/ml. Data points represent values from a single replicate or the average of two replicates (as reported in Table 2 in the main text), with error bars corresponding to the standard deviation. In panel (a), CFU/ml data are also shown for the plasmid-based $W_{\Delta LFP}$-pL13 strain, used as a reference.

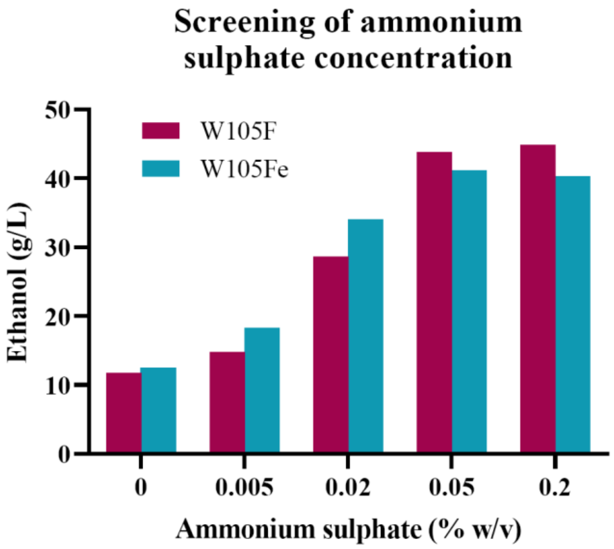

Figure S3. Fermentation performance screening in CWP + PIPES in test tube experiments for the W105F and W105Fe strains with different concentrations of ammonium sulphate. Bars indicate the ethanol concentrations of single replicates after 72 h.

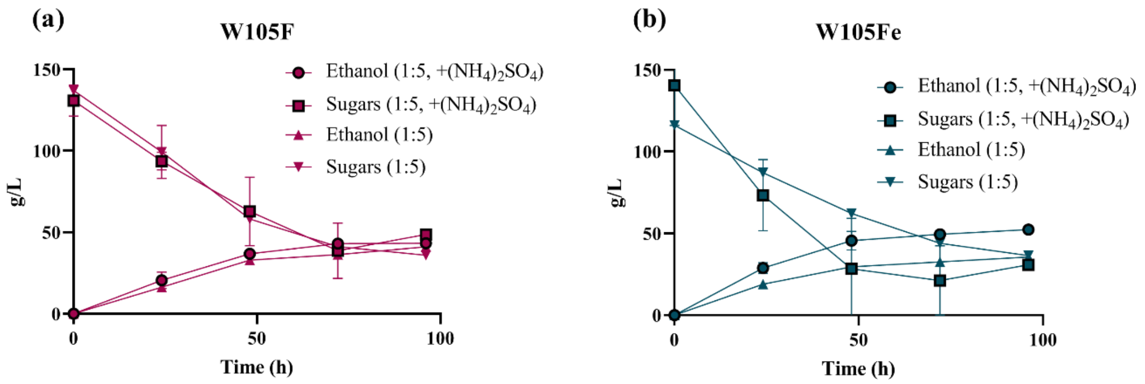

Figure S4. Performance comparison of W105F (a) or W105Fe (b) between the ammonium sulphate-supplemented CWP and non-supplemented CWP conditions, all of them with 1:5 inoculum ratio. Fermentation time course data from pH-controlled bioreactor experiments are represented as concentration time series of sugars and ethanol. The reported profiles are also available in Figure 1 and Figure 3 of the main text, and are herein shown in the same plots to support pairwise comparison. Data points represent values from a single replicate or the average of two replicates (as reported in Table 2), with error bars corresponding to the standard deviation.
